# Anoikis in phenotypic reprogramming of the prostate tumor microenvironment

**DOI:** 10.3389/fendo.2023.1160267

**Published:** 2023-04-05

**Authors:** Prerna R. Nepali, Natasha Kyprianou

**Affiliations:** ^1^Department of Urology, Icahn School of Medicine at Mount Sinai, New York, NY, United States; ^2^Department of Oncological Sciences, Icahn School of Medicine at Mount Sinai, New York, NY, United States; ^3^Department of Pathology and Cell-Based Medicine, Icahn School of Medicine at Mount Sinai, New York, NY, United States

**Keywords:** prostate cancer, anoikis, treatment resistance, EMT, metastasis, phenotypic reprogramming, extracellular matrix

## Abstract

Prostate cancer is one of the most common malignancies in males wherein 1 in 8 men are diagnosed with this disease in their lifetime. The urgency to find novel therapeutic interventions is associated with high treatment resistance and mortality rates associated with castration-resistant prostate cancer. Anoikis is an apoptotic phenomenon for normal epithelial or endothelial cells that have lost their attachment to the extracellular matrix (ECM). Tumor cells that lose their connection to the ECM can die via apoptosis or survive via anoikis resistance and thus escaping to distant organs for metastatic progression. This review discusses the recent advances made in our understanding of the signaling effectors of anoikis in prostate cancer and the approaches to translate these mechanistic insights into therapeutic benefits for reducing lethal disease outcomes (by overcoming anoikis resistance). The prostate tumor microenvironment is a highly dynamic landscape wherein the balance between androgen signaling, cell lineage changes, epithelial-mesenchymal transition (EMT), extracellular matrix interactions, actin cytoskeleton remodeling as well as metabolic changes, confer anoikis resistance and metastatic spread. Thus, these mechanisms also offer unique molecular treatment signatures, exploitation of which can prime prostate tumors to anoikis induction with a high translational significance.

## Introduction

Prostate cancer (PCa) is a heterogeneous disease wherein prostatic intraepithelial neoplasia progresses into invasive metastatic carcinoma (1, 2). Treatment modalities effectively used in the clinic for localized disease include prostatectomy, radiation therapy and medical or surgical androgen deprivation (3). PCa is treatable if diagnosed early, however the high mortality rate associated with the disease is a result of therapeutic resistance to androgen-deprivation therapy (ADT), radiotherapy and chemotherapy. Targeted therapeutics that can overcome resistance are being developed by determining the underlying mechanisms of androgen receptor (AR) signaling in PCa. Moreover, distant metastasis associated with lethal disease holds high potential for the identification of new actionable sites during invasion and progression of PCa cells.

Eukaryotic cells die *via* mechanisms inducing apoptosis, anoikis and necrosis (4, 5). Normal tissue homeostasis is a result of a balance between cell proliferation and apoptosis (6). Apoptosis is the main pathway for tumor cell death in response to treatment modalities in prostate cancer patients and defects in apoptosis have been linked to not only tumor progression and metastasis but also therapeutic resistance (7, 8). The term “anoikis” (Greek for “homelessness”) was coined by Frisch to define apoptotic cell death induced by insufficient cell-extracellular matrix (ECM) interactions (1, 9). Anoikis prevents dissemination of cells to inappropriate distant sites during metastasis (1, 10). Resistance of cancer cells to anoikis confers them increased survival in the absence of ECM attachment, ability to travel through circulatory and lymphatic systems followed by dissemination and attachment at secondary sites leading to metastasis (11, 12). Understanding the molecular mechanisms contributing to anoikis resistance is thus crucial for development of targeted therapeutics (11).

### Mechanisms of anoikis induction

Mechanistic regulation of anoikis proceeds by cell death receptor-mediated (extrinsic) and mitochondrial (intrinsic) apoptotic pathways (13). Death ligands (FAS, Tumor Necrosis Factor-a, TRAIL) bind to the extracellular domain of the death receptors to activate the death receptor pathway (14, 15). The death-inducing signaling complex (DISC) is formed when death effector domain (DED) of FAS-associated death domain (FADD) binds with caspase-8 (FLICE). FLICE when released into the cytoplasm cleaves caspase-3 and caspase-7 followed by cell death of cellular substrates (12, 16). FLICE-inhibitory protein (FLIP) inhibits caspase-8 binding and activation by having preferential affinity for DISC. This death receptor pathway wherein matrix attachment prevents FAS-induced apoptosis is mechanistically engaged in anoikis induction (12). Loss of ECM anchorage leads to increased FAS, decreased FLIP leading to anoikis (17, 18). X-linked inhibitor of apoptosis proteins (XIAP) binds and inhibits active Caspases 3/7. Anoikis resistant PCa cells have increased levels of XIAP compared to normal prostate epithelial cells (14, 19). In addition, increased level of XIAP in metastatic PCa cells is associated with anoikis resistance (14, 20). The mitochondrial intrinsic apoptotic signaling is regulated by proteins in the Bcl-2 family (15). Tumor cell survival and apoptosis is regulated by the balance between pro-apototic Bcl-2 proteins (Bax, Bad, and Bid) and anti-apoptotic proteins (Bcl-2 and Bcl-xL) (15). Initiation of death signals leads to the translocation of Bax or Bid from the cytosol to the outer mitochondrial membrane leading to cytochrome release and caspase 9 and 3 activation (12). This translocation of Bid after loss of adhesion has been observed during anoikis of mammary epithelial cells (12). Bcl-2 binds to Bax and Bad, maintains mitochondrial membrane integrity and prevents apoptosis. In Ras-transformed intestinal cells, decreased Bak and failure to downregulate Bcl-xL leads to anoikis resistance. Non-transformed intestinal cells undergo anoikis *via* release of mitochondrial Omi/HtrA2 and detachment-induced down-regulation of Bcl- xL (14). Moreover, there is evidence to suggest the presence of Bcl-2-independent anoikis mechanisms in PCa cells (21). The pro-apoptotic signal of Bim (Bax activator) is muted by its translocation to the mitochondria and interaction with Bcl-xL after cell detachment. Upregulation of Bim *via* inhibition of Src, Erk and Akt pathways “primes” breast cancer cells to anoikis (22).

Bit1 protein (Bcl2 inhibitor of transcription 1) mechanistically mediates anoikis *via* integrins, independent of caspases (23, 24). Bit1 apoptosis is blocked by integrin-mediated attachment thus making Bit1 a crucial player in anoikis. Bit1 suppression has been correlated with advanced metastasis *via* Erk pathway stimulation and anoikis resistance in breast cancer models (24). Bit1 expression is reduced in non-small cell lung carcinoma tumors and exogenous Bit1 overcomes anoikis resistance in human lung adenocarcinoma cells *via* caspase-independent pathways by prohibiting anchorage-independent growth (13). Bit1 overexpression/induction can be a critical path in overcoming anoikis resistance in PCa treatment.

## Phenotypic landscape of prostate tumor microenvironment

### Epithelial-mesenchymal transition

**Figure 1:**
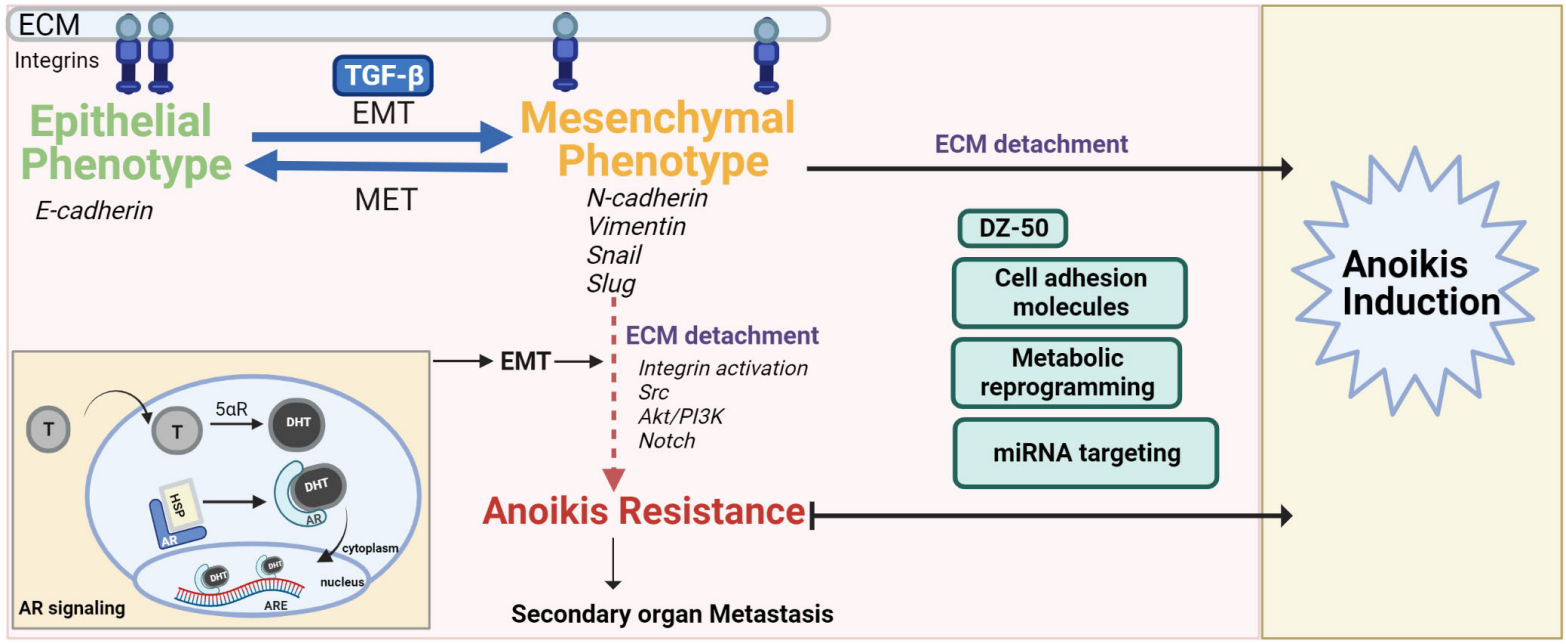
The interplay of EMT-MET maintains the pivotal balance between normal cell anoikis and cancer progression/anoikis resistance. The mesenchymal phenotype contributes to anoikis resistance after ECM detachment *via* activation/dysregulation of pathways such as androgen signaling, Akt/PI3K, Src and Notch signaling. Anoikis resistance can be overcome by drugs (DZ-50), regulating ROS balance, targeting miRNA or cell adhesion molecules that inactivate these pathways, push the switch in favor of MET and/or make the cancer cells anoikis sensitive.

Epithelial-mesenchymal transition (EMT) is a highly conserved cellular process that contributes to physiologic and pathologic conditions and allows a polarized epithelial cell to adopt a mesenchymal phenotype (25). The polarized epithelial cell thus loses its ability to maintain interactions with the basement membrane and converges into an apoptosis-resistant, migratory and invasive mesenchymal cell. Several studies have shown that the malignant transformation of cells is a result of disruption of homeostasis by increased EMT (25). Cellular markers for the morphological profiling of EMT include epithelial markers (E-cadherin, B-catenin, Cytokeratin) or mesenchymal markers (N-cadherin, Vimentin, Fibronectin, Snail, Slug, Twist or Matrix Metalloproteinases-2,3,9) (25) (**Figure 1**). The phenotypic landscape of EMT is represented by loss of epithelial markers (E-cadherin, B-catenin, occludin) and a gain of mesenchymal markers (e.g. N-Cadherin, Zeb-1/2, vimentin and E-cadherin repressors such as Snail, Twist) (4). Ras is also a critical signaling molecule which induces EMT *via* the PI3K or MAPK pathways (6, 26, 27). Loss of E-cadherin expression leads to loss of adherence to junctional proteins (B-catenin and actin), ultimately compromising epithelial cell plasticity and conferring anoikis resistance (4). Phenotypic EMT functionally contributes to tumor progression and recurrence *via* enhanced invasive and metastatic properties, increase in cancer stem cell populations and therapeutic resistance consequential to anoikis resistance (28).

The multilayered and complex relationship between the signaling effectors of EMT and anoikis in cancer progression and therapeutic resistance, remains the focus of intense investigative efforts. Studies in breast cancer using conditional knockout of E-cadherin have shown correlation of E-cadherin knockdown to a highly metastatic, angiogenesis- dependent anoikis resistant phenotype (29). Interestingly, anoikis sensitivity was restored in mammary epithelial cells when E-cadherin and B-catenin were knocked down together (4, 28, 30). Ankyrin-G is an actin cytoskeleton and cell membrane linker protein. The interaction of Ankyrin-G with E-cadherin also influences anoikis resistance. EMT downregulates ankyrin G leading to reduced p14ARF (tumor suppressor and anoikis promoter) (4, 31). Vimentin, a hallmark of mesenchymal cells is an intermediary filament that provides structural support to cells and is overexpressed in human cancers including prostate cancer. A significant association has been found between vimentin’s regulation and decreased prostate tumor cell invasiveness (32–36). Compelling evidence linking anoikis and EMT stems from studies on prostate apoptosis response-4 (Par-4), a tumor suppressor which selectively induces apoptosis *via* caspase-dependent mechanisms in cancer cells but not normal cells (37). At the mechanistic level, Arylquin 1 (a 3-arylquinoline derivative and potent Par-4 secretagogue) binds to vimentin, displaces Par-4 from vimentin and the secreted Par-4 induces paracrine tumor cell apoptosis in PCa cells (37, 38). Ginsenoside 20(R)-Rg3 can prevent EMT induction *via* p38 MAPK inhibition, vimentin and Snail suppression, E-cadherin increase and by overcoming anoikis resistance, migration and invasion in lung cancer cells (39). As the mesenchymal phenotype confers resistance to anoikis induction, the therapeutic response can be improved by priming them into being reprogrammed to the epithelial phenotype [reversing the EMT phenotype to mesenchymal-epithelial transition (MET)] (4). The plasticity of prostate cells in the tumor microenvironment (TME) allows for the interconversion between de-differentiated EMT and re-differentiated MET phenotypes during tumor progression (40). Transcriptional repressors of E-cadherin, such as Snail, activate the PI3K/Akt survival pathway, inhibiting capsase-3-mediated apoptosis (1, 4). Twist is another transcriptional regulator of EMT by inducing mesenchymal markers such as fibronectin and N-cadherin (1). Twist plays an anti-apoptotic role and its loss could be critical in conferring anoikis sensitivity in cancer cells (1, 41, 42). The zinc finger E-box-binding homeobox 1 (Zeb) family of transcription factors, also play a role in cancer progression wherein Zeb1 and Zeb2 suppress E-cadherin transcription (43). In studies using kidney cell line, the plasticity balance between epithelial to mesenchymal state interconversion is regulated by transforming growth factor-beta (TGF-β) related ZEB expression and miR-200 negative correlation (44). Knocking down Zeb1 as a mode of overcoming anoikis resistance and improving therapeutic response provides promise, wherein silencing Zeb1 in PCa cell lines leads to decreased cancer stem cell markers (CD44, CD133 and SOX2) and decreased ability to form protospheres and colonies (45). Of translational significance is the association of Zeb1 overexpression in highly aggressive tumors and higher Gleason grade in PCa, implicating a role for this EMT molecular regulator in prostate tumor progression to advanced disease (46).

TGF-β is a multifactorial regulator peptide that plays a dual role as an inhibitor of tumorigenesis in normal and early stage disease and a promoter of advanced PCa (32). In early tumor development, TGF-β induces cell cycle arrest and apoptosis to inhibit tumor development. This is then followed in the later stages by TGF-β induced tumor promotion through aberrant cell cycle regulation (47). In metastatic disease, increased production of TGF-β is associated with immunosuppression *via* T-cell regulation, ECM degradation, EMT propagation and angiogenesis – a combination that positively supports tumor cell invasion. TGF-β induces tumor progression and metastasis by promoting EMT in PCa cells (32, 48) *via* constitutively activating Akt and thus inhibiting SMAD3 translocation to the nucleus (49, 50). At the same time, there is a complex and fateful interaction between TGF-β and tumors making it imperative to target TGF-β before its involvement in the metastatic process, while it switches from a tumor suppressor to metastasis promoter (48). TGF-β induced EMT in PC3 PCa cells was inhibited *via* nuclear factor κB (NF-κB) signaling blockade resulting into decreased vimentin expression (32, 51). Snail regulates TGF-βI expression and TGF-β promotes post-translational modification of Snail (sumoylation). Sumoylated Snail leads to increased invasiveness and aggressiveness in PCa cells *via* Hes1 (transcriptional target in Notch signaling cascade) (52). Studies focused on understanding the effects of systemic TGF-β inhibition on early stages of prostate tumorigenesis have used trivalent TGF-β receptor trap (RER) comprised of domains from the TGF-β II and III receptors to completely block (a) TGF-β II binding and (b) TGF-β1 and TGF-β3 signaling in cultured PCa cells. Early inhibition of TGF-β using RER led to reduced prostate cell proliferation (reduced Ki67 positive cells) and invasion capacity of cells in prostate glands of Pten conditional null mice (53).

### Androgen signaling contributing to prostate anoikis

Prostate glandular epithelial cells depend on androgens for growth and survival. The AR is a steroid receptor that stays inactive while bound to chaperone proteins (heat shock protein 90) in the cytoplasm. Testosterone dissociates from sex hormone binding protein, diffuses across the prostatic plasma membrane and is converted into dihydrotestosterone (DHT) by cytochrome p450 enzyme 5α-reductase. AR after binding to DHT undergoes a conformational change and translocates into the nucleus to bind androgen response elements (ARE) leading to the stimulation of androgen-dependent proteins (54–58) (**Figure 1**). ADT achieved surgically or chemically (with luteinizing hormone-releasing hormone (LHRH) agonists, LHRH antagonists, or anti-androgens) is the mainstay therapeutic modality for the treatment of advanced PCa. AR is not only a driver of PCa progression but is also critical in clinical response to therapy (59). Significantly enough, androgen signaling has been implicated in EMT-MET phenotypic interconversions (60, 61). The evidence points to negative correlation between EMT effectors and AR activity, co-involved in metastatic PCa progression (62). AR deletion in the Transgenic Adenocarcinoma Mouse Prostate (TRAMP) tumors leads to TGF-β1 dependent decrease in MET, increased mesenchymal state and EMT also followed by higher cell invasion rate and anoikis resistance (63). AR expression is increased in triple negative breast cancer and this increase and anchorage-independent survival/anoikis resistance is also TGF-β dependent (64, 65). Interestingly, TGF-β mediated apoptosis was enhanced rather than prevented by androgens (DHT) in hormone-sensitive PCa cells thus opening avenues for priming PCa cells to apoptotic induction pathways by using physiologic levels of androgens (66). Bcl-2 (apoptotic suppressor) is able to block the apoptotic signaling of TGF-β and DHT. Thus, Bcl-2 has a role in the emergence of castration resistant PCa (CRPC) (67). Pre-clinical studies have shown that resistance to cabazitaxel chemotherapy can be overcome by anti-androgen induced EMT to MET conversion in androgen sensitive tumors (68). Interestingly enough a dual role for AR has been shown in the promotion of hepatocellular carcinoma (HCC) initiation; and loss of AR leads to HCC metastasis with increased incidence of undifferentiated tumors in preclinical models (69). Hepatic AR suppressed p38 phosphorylation and NF-κB/matrix metallopeptidase 9 pathway leading to anoikis induction and reduced migration of HCC cells (69). Thus AR plays a role in the phenotypic landscape of the TME in other cancers, besides prostate tumors.

The prostate epithelium consists of luminal, basal and neuroendocrine cells wherein the former two possess progenitor activity (70). Deletion of E-cadherin in prostate luminal cells results into anoikis followed by a tissue repair program of differentiation of basal epithelial cells to luminal cells (70). Basal cells express adhesion -associated membrane receptors and their substrates in the ECM and can establish cell-matrix interactions autonomously thus escaping anoikis (71). Transmembrane serine protease 4 (TMPRSS4) is responsible for invasion and proliferation of PCa cells and its elevated levels are associated with poor prognosis in several cancers including prostate, gastric, colorectal and non-small cell lung cancer. Stemness-related factors such as SOX2, BMI1 and CD133 can functionally promote anoikis resistance. The stemness and anoikis resistance promoting properties of TMPRSS4 were also associated with contributions by EMT-inducing transcription factors Slug and Twist (72). Metastatic human PCa cells when exposed to clinically-relevant radiation doses lead to generation of two radiation-surviving cell populations- adherent senescent-like cells and non-adherent anoikis-resistant stem cell-like cells (73). The anoikis-resistant cells also demonstrate radioresistance-associated EMT, *via* Snail over-expression and activation of the PI3K/Akt and Erk1/2 pathways (73).

Src is highly expressed in PCa and its activation has been associated with cancer progression and anoikis resistance (15). Dasatinib, a Src family kinase (SFK) inhibitor shows therapeutic promise in controlling tumor growth and lymph node metastases in PCa models *in vivo* (15, 74). Dasatinib blocks Focal adhesion kinase (FAK) in human PCa cells (15, 75). Persistent redox signals in PC3 cells result into chronic pro-survival signaling *via* Src oxidation in the absence of cell adhesion leading to epidermal growth factor phosphorylation (76). Notch activation induces anoikis resistance of prostate luminal progenitors by augmenting NF-kB activity and thus rescuing their capacity for renewal and unipotent differentiation (71). NF-κB also leads to anoikis resistance and tumor cell survival downstream of the PI3K/Akt pathway (11, 77).

The new stromal microenvironment, formed as a result of tumor cell invasion into basement membrane, is comprised of increased ECM remodeling, angiogenesis, growth factor bioavailability, protease activity and inflammatory influx (1). Anoikis resistance conferred by loss of apoptotic pathways and activation of survival mechanisms, is followed by phenotypic changes and increased neovascularization within the reactive stroma in the prostate TME (1). The myofibroblasts in the reactive stroma secrete molecules fibronectin, collagen I and III, glycoproteins and proteoglycans that directly influence ECM remodeling (1). Thus the reactive stroma is responsible for initiating EMT and prostate tumor neovascularization (1). Connective tissue growth factor (CTGF) is expressed by stromal cell types such as endothelial cells, fibroblasts, smooth muscle cells and myofibroblasts. CTGF plays a role in (a) stromal extracellular matrix synthesis, proliferation and migration in smooth muscle cells; (b) proliferation, cell adhesion, cell spreading in fibroblasts and also (c) endothelial cell adhesion, proliferation, migration, and angiogenesis. Thus, CTGF is a key component of the reactive stromal compartment of different cancers including tumor-promoting prostate stromal cells. TGF-β1 is a critical regulator of CTGF in the reactive prostate stroma (78). The expression levels of CTGF can influence therapeutic response (79). Over expression of CTGF is associated with poor clinical outcomes including advanced disease with larger tumor size, worse metastasis, decreased treatment response in many cancers such as but not limited to breast, gastric, esophageal, pancreatic and prostate cancer (79, 80). Interestingly, in lung cancer CTGF displays an anti-metastasis function wherein it binds to epidermal growth factor receptor (EGFR) leading to EGFR degradation by ubiquitination and also suppresses the phosphorylation of c-Src and promotes anoikis (81). This potential anoikis-promoting mechanism and metastasis suppressive impact of CTGF with anti-EGFR therapy serve as a therapeutic avenue for lung cancer (81).

### The extracellular matrix and actin cytoskeleton

Progression of PCa is a dynamic integration of three primary events that critically impact the TME landscape: Phenotypic EMT, formation of a reactive stroma which involves structural rearrangement of the ECM, and cell death *via* anoikis (1). Diverse protein kinases and phosphatases are protagonists of anoikis regulation (6). The transmembrane proteins- integrins are primary regulators of cell-ECM interactions (15). FAK is an integrin signaling molecule that is recruited into focal adhesions upon cell-ECM contact, leading to phosphorylation and activation of FAK (11). Mechanistically, anoikis resistance is a result of constitutive activation of FAK (82, 83). PI3K is a FAK-activated protein that recruits Protein Kinase B (PKB/Akt) leading to cell survival (11). This complex chain of integrin-dependent events have been shown to be involved in PCa progression to metastatic disease (15, 84). Src belonging to the SFK is activated *via* β1-integrin through FAK in the focal adhesion complex upon cell-ECM adhesion (15). Anoikis resistance in PCa has been linked to the phosphorylation of FAK and Akt by β1-integrin (16, 85). Notably, loss of β1-integrins in PCa cells overcomes anoikis resistance and treatment with the β1-integrin neutralizing antibody mAB 33B6 reduces tumor metastases *in vivo* (85). Collagen XIII is an integrin-binding transmembrane protein responsible for cell-cell and cell-matrix interactions, that induces β1-integrin activation, cancer cell stemness, metastasis and anoikis resistance (86). Rad9, a DNA damage response and repair protein is upregulated in human PCa. Rad9 contributes to tumor survival, proliferation, migration and anoikis resistance in PCa cells, a phenomenon that can be overcome by silencing Rad9, towards increasing anoikis sensitivity (87). αvβ3 integrin can confer anoikis resistance and a migratory phenotype to PCa cells (11). Invasive AR-negative, androgen-independent PC3 cells express αvβ3 integrin whereas noninvasive androgen-sensitive LNCaP cells do not (88). Members of the arginine-glycine-aspartic acid (RGD)- binding integrin family that recognize the RGD sequence in the ECM are expressed differentially in primary prostate tumors compared to normal prostate tissue and are interesting therapeutic targets (89). Studies using Small-molecule integrin antagonists against RGD-integrins obstruct glioblastoma infiltration and malignancy *via* detachment-mediated anoikis induction in glioma cancer stem cells (90). The upregulation of adhesion complex protein-Talin is associated with anoikis resistance and increased invasion and metastatic properties in prostate tumor cells followed by downstream activation of FAK/Akt signaling. Human PCa specimens exhibit a higher expression of cytoplasmic talin1 in metastatic tissue compared to primary tumors (83, 91); and a significant inverse correlation between talin and E-cadherin in human prostate tumors and metastatic lesions has been reported (91). Talin thus serves as an anoikis effector which can be therapeutically targeted within the prostate TME and vascularity (83). The humanized monoclonal antibody Trastuzumab that targets human EGFR2 suppresses the angiogenic and invasive properties of anoikis-resistant endothelial cells. The reversal of tumor phenotype by Trastuzumab is *via* reduction in the expression of Heparan sulfate proteoglycans such as syndecan‐4 and perlecan that play a role in proliferative-migratory and angiogenic pathways respectively and are also overexpressed in anoikis-resistant endothelial cells (92, 93).

β-catenin is an adhesion molecule that functionally interacts with E-cadherin and regulates the dynamics of the actin cytoskeleton remodeling and phenotypic EMT dictating cell behavior (25). When invasive cells undergo EMT, B-catenin remains in the cytosol and nucleus in association with an activated Wnt pathway in comparison to non-invasive cells wherein it is on the membrane (25). B-catenin is a contributor to an invasive and malignant phenotype by binding to AR and translocating into the nucleus. This effect of B-catenin and AR interaction is further amplified in cells lacking E-cadherin (EMT phenotype) (25, 94, 95), pointing to B-catenin as a potential predictive biomarker for PCa progression and therapeutic response (25, 96). Tyrosine kinase inhibitor Imatinib mesylate, which is clinically used to treat leukemia and gastric cancers, might be considered for potentially treating PCa owing to its inhibition of the Wnt/B-catenin signaling pathway (35, 97, 98).

CTGF is a target gene for Yes-associated protein (YAP) which is an organ size regulator and a human oncogene. The Hippo tumor suppressor pathway is activated upon cytoskeletal reorganization and cell detachment and phosphorylates and inhibits YAP leading to anoikis in non-transformed cells. The Hippo pathway is deregulated in PCa cells and the expression of its kinases Lats1/2 is downregulated in metastatic PCa leading to anoikis resistance (99). YAP knockdown or inhibition by small molecule inhibitor (CA3) of YAP transcriptional activity overcomes anoikis resistance in melanoma cells (100). The β-adrenergic receptor antagonist, propranolol was able to overcome neuroendocrine signaling in cervical cancer cells by prohibiting norepinephrine-induced YAP-mediated anoikis resistance (101). Decreased α3-integrin expression is associated with cancer progression to metastasis under detached and low anchorage conditions *via* YAP upregulation (102). In the same PCa model, Abl kinase that plays a role in cytoskeleton remodeling in response to extracellular stimuli, cell motility and adhesion seemed to work together with α3-integrin to suppress RhoA activity and support Hippo function and thus restrain metastasis (102). Abl kinase inhibitor and Platelet-derived growth factor targeting imatinib shows cancer cell proliferation in PDGF independent tumors (102–104). Understanding the Hippo signaling pathway and the intricacies of its regulators in the context of anoikis are necessary for therapeutic development of Hippo and anoikis-targeted treatment in PCa.

## Therapeutic value of anoikis

Overcoming resistance in aggressive PCa needs to be a combination of (i) reversing anoikis resistance in tumor cells by increasing susceptibility to anoikis-inducing agents (ii) blocking key players or pathways involved in cancer cell seeding and survival at secondary locations (iii) making tumor cells less sensitive to chemotactic cues of the new target organ, thus decreasing the probability of metastatic spread (1).

### Novel quinazoline-based compounds

α1-adrenoreceptor antagonists are clinically approved for the treatment of hypertension, and as first-line treatment for benign prostatic hyperplasia and show anti-tumor and pro-apoptotic activity (3, 105, 106). Clinically available quinazoline-based α1-adrenoceptor antagonists such as doxazosin and terazosin induce TGF-β dependent apoptosis in the epithelial, endothelial and smooth muscle cells of the prostate (15, 107). Doxazosin induces apoptosis by activating caspase-3 followed by FAK cleavage and ultimately anoikis thus operating *via* an α1-adrenoreceptor independent, anoikis dependent, anti-angiogenetic mechanism (108–111).

Structural optimization of quinazoline-based α1-adrenoceptor antagonist, Doxazosin, has led to the generation of a compound namely DZ-50 (16, 112, 113). DZ-50 induced anoikis and anti-angiogenesis in PCa and human PCa xenografts in nude mice (4). Critical effectors of EMT (N-cadherin) and tight junctions that are associated with poor clinical outcomes in PCa are downregulated during DZ-50 induced anoikis (114, 115). DZ-50 and its property to induce EMT to MET transition can thus be leveraged for metastatic CRPC treatment and potentially overcoming therapeutic resistance *via* an anoikis-driven response. Insulin growth factor binding protein 3 which is involved in stromal remodeling during PCa progression is downregulated in DZ-50 treated PCa cell lines (114). Genome-wide analysis in the DU-145 human PCa cell line has shown that DZ-50 down regulates target genes involved in focal adhesion integrity (fibronectin, integrin-a6 and talin), tight junction formation (claudin11) as well as angiogenesis modulator thrombospondin 1 (113). Targeting the TGF-β signaling pathway has also been addressed *via* these quinazoline based α1-adrenoceptor antagonists or anti-sense inhibition of TGF-β1 expression (107, 116).

### Targeting TGF-β signaling

Small molecule inhibitors for TGFβR kinases are under investigation in clinical trials for treatment of cancer patients *via* TGF-β signaling targeting. Galunisertib which selectively binds to TGFβR1 and inhibits its kinase activity has shown pre-clinical promise and Phase 1/2 clinical trials are underway for its use in metastatic disease in a variety of cancers including prostate, pancreatic cancer and glioblastoma (117). Studies conducted on Dominant Negative TGFβRII male mice using combination of galunisertib and FDA-approved antiandrogen enzalutamide, not only showed reduced prostate tumor growth by increased apoptosis and reduced tumor cell proliferation but also reduced cofilin and reversion of EMT to MET phenotype (118). Phase 2 clinical trials are underway for the use of Galunisertib and Enzalutamide in Metastatic CRPC (NCT02452008). Chemotherapy-induced transcriptome reveals a highly activated TGF-β signaling, involving a chemotherapeutic associated stimulation of Smad2/3 phosphorylation, cell migration, and upregulation of EMT and cancer stem cell markers. Herein, RER with cisplatin showed improved tumor inhibition in xenograft ovarian cancer models thus focusing on avenues of combining chemotherapeutics with TGF-β inhibition (119). Proteolysis-targeting chimeras (PROTACs) are bifunctional molecules that recruit a target protein and bind it to ubiquitin ligase leading to binding of the protein to ubiquitin and resulting into its degradation (43). PROTAC molecules were developed for targeting AR wherein ARV-110 (oral PROTAC drug for AR degradation) went into Phase 1 clinical trials for patients with metastatic CRPC (120). PROTAC technology shows promise in potentially overcoming anoikis resistance (43).

### Death finds a pathway

The death receptor pathway inhibitor- FLIP has been demonstrated to assist malignant cells in escaping anoikis (12, 14). FLIP inhibition with siRNA or small molecules in a mouse model of PCa metastases prevented the formation of distant metastatic tumors (14). In studies designed to use a novel high-throughput screen to identify molecules that can sensitize resistant cancer cells to anoikis, it was seen that anisomycin was able to sensitize anoikis-resistant PCa cells by decreasing levels of FLIP and thus activating the death receptor pathway (121). Polyphenylurea based inhibitors of XIAP have shown to sensitize resistant PCa cells to anoikis as well as prevent distant tumor formation in orthotopic PCa models (14, 20, 122, 123).

### Metabolic reprogramming

Reactive oxygen species (ROS) have a dual effect on anoikis resistance based upon the cell type and the mechanisms are not properly understood (124–126). Transient changes in levels of ROS are essential in cell cycle regulation, cell proliferation and adhesion (76). However, it was recently reported that ROS production is elevated in cancer cells (127), with evidence supporting a connection between elevated ROS in metastatic prostate carcinoma cells and resistance to anoikis *via* chronic pro-survival signaling (76). Tumor cells themselves also trigger anti-oxidant enzymes for survival in response to elevated ROS, by inducing transcription factor Nrf-2 (11). Snail is involved in increased ROS production in PCa cell lines *via* EMT (128). The differences in the apoptotic mechanisms in PCa cells and their variant antioxidant system has been implicated in cisplatin resistance (129). PC3 cells cope with stress conditions *via* decreased p53 and Bax to become resistant to apoptotic regulatory mechanisms, cell cycle arrest and cytostatics (129). The receptor tyrosine kinase- EGFR when phosphorylated by Src in high ROS conditions has been shown to induce anoikis inhibition in PCa cells (76, 130, 131). In lung cancer, anoikis resistance could be overcome by NOX4 knockdown followed by reduced activation of Src and EGFR (124). Further studies establishing a strong link between ROS and anoikis resistance show that when PC3 cells are detached, there is an increase in the expression of leukotriene B_4_ receptor-2 (BLT_2_), causing a cascade of events- NOX activation, ROS generation, NF-κB activation downstream of BLT_2._ This BLT2-NOX-ROS-NF-κB axis thus plays a role in incurring anoikis resistance in PCa cells and serves as a novel therapeutic target to overcome resistance (132). ROS generating agent plumbagin overcomes anoikis resistance *via* the inhibition of NF-kB in resistant breast cancer cells (133). In mammary epithelial cells, a role of anti-oxidation agents has been implicated, in cancer cell survival and anchorage-independent colony formation (134). Androgen-independent human prostate cancer PC3 cells have increased antioxidant capacity *via* increased metallothionein expression and decreased pro-apoptotic genes thus resulting into cisplatin resistance (129). In sharp contrast, plumbagin is lethal to prostate cancer cells compared to cisplatin, possibly through ROS generation that overcomes anoikis resistance (135). Certain antioxidants may also act as tumor promoters *via* anoikis inhibition (28, 134). Thus, understanding the antioxidant profile at different stages of PCa may elucidate new mechanisms for overcoming anoikis resistance and improving therapeutic vulnerability in advanced tumors.

In the normal epithelium, cell detachment from the ECM is associated with impaired glucose transport, ATP deficiency followed by apoptosis. In metastatic cancer cells however, survival and anoikis resistance are consequences of high-energy generation through increased oncogene products (2, 124). Interestingly enough, there is evidence to suggest disruption of anoikis resistance and metabolic reprogramming as a therapeutic strategy for treatment of advanced PCa (76). Cell migration-inducing protein (CEMIP) plays a role in migration, invasion, pyruvate and lactate production, ATP level regulation as well as detachment-induced anoikis (2). CEMIP is overexpressed in PCa-AR cells and promotes tumor metastasis *via* metabolic reprograming (2). Furthermore, CEMIP causes anoikis resistance and increases metastatic potential of cells by inducing PKCa translocation *via* calcium leakage from the endoplasmic reticulum. The plasma membrane located PKCa induced protective autophagy through Bcl-2/Beclin complex dissociation causing survival of the ECM-detached cells (136). CEMIP is elevated in late stage PCa tissue as compared to precancerous tissue and its suppression can be used to impair EMT and metastasis by inducing anoikis (136).

In human mammary cells, upregulation of pyruvate dehydrogenase kinase 4 (PDK4) through estrogen-related receptor gamma causes reprogramming of glucose metabolism by reducing the conversion of glycolysis-derived pyruvate into acetyl CoA leading to anoikis resistance *via* decreased glucose oxidation (137, 138). Microarray analysis has revealed elevated PDK4 in human cancer cell lines including prostate (DU145), renal, ovarian and lung (137). PDK4 along with other metabolic markers such as PGK1 and glucose-6-phosphate dehydrogenase hold tremendous promise as glucose metabolic biomarkers in circulating tumor cells during PCa metastasis (139). Mitochondrial DNA alterations detected in PCa cells are linked to anoikis resistance *via* the PI3K/Akt signaling (140).

### MicroRNA in anoikis

An increasing body of evidence has established the role of miRNAs in PCa tumorigenesis, anoikis/apoptosis avoidance and associated metastasis. miRNAs are interesting candidates not only as biomarkers of metastatic disease but also potential targets for therapeutic intervention. Anoikis resistance in PCa and bone metastasis has been shown to be a result of activation of the PI3K/Akt pathway and downregulation of miR-133a-3p (27, 141). Circular RNA (Circ_0004585) binds with miR-1248 and enhances PCa invasion, anoikis resistance and metastasis (142). Growth factor granulin is highly expressed in a variety of cancers and has pro-carcinogenic effects such as increased epithelial cell division and anoikis resistance (143–145). Granulin facilitates migration and anchorage-independent growth in androgen dependent and independent PCa cell lines (144, 146). miR107 has been shown to downregulate granulin expression in PCa cells, thus implicating this group of miRNA as therapeutic targets to overcome anoikis resistance (145). Elevated levels of miRNA (miR-16, miR-148a and miR-195) involved in the PI3K/Akt pathway, have been detected in the plasma of PCa patients (147). miR-4534, is overexpressed in PCa and functionally contributes to downregulation of *Pten* tumor suppressor (148). Furthermore, inhibition of MiR-186-5p also resulted into reduced anoikis resistance and survival in PC-3 and/or MDA-PCa-2b PCa cells *via* inhibition of PI3K/Akt signaling (149, 150).

## Future directions

Anoikis resistance contributes to progression of advanced metastatic PCa and emergence of therapeutic resistance. The ideal treatment modality for PCa would be to build a patient-specific profile that predicts the nature of individual tumors including metastatic potential using pathological grading along-with anoikis-related signatures to predict the treatment response of specific type of tumors. There is rapidly growing evidence on the mechanisms of anoikis and its translational significance during prostate cancer progression (as predictive biomarker anoikis-centered signatures, and therapeutic targeting platform for advanced metastatic tumors) and recurrent disease. However, there are significant gaps in our knowledge about exploiting the dynamics of EMT-MET within the TME. The phenotypic EMT and its interconversion to MET and consequential decreased resistance can be navigated by anoikis sensitizing agents to enable the development of new targeted therapeutics against lethal disease (**Figure 1**). Novel quinazoline compounds which disrupt FAK and their interactions with proteins such as Bit1 (only released after loss of integrin-ECM interactions) will help elucidate the complexities of anoikis resistance and identify novel therapeutic targets in caspase-independent apoptotic pathways (3, 13). While compounds such as DZ-50 are able to induce anoikis *via* EMT to MET cycling their potential use in combination therapy with taxanes to overcome chemotherapeutic resistance in PCa treatment remains a matter of further investigation. Bit1 expression in the TME versus in normal prostate cells and its regulation of anoikis in the prostate during normal cell physiology requires in depth assessment to harvest its therapeutic potential (151). The Bcl-2 family of proteins and their role in anoikis regulation/resistance for PCa treatment require further mechanistic insight to develop specific treatment strategies. Brain-derived neurotrophic factor (BDNF) is overexpressed in many human cancers including PCa. BDNF activation leads to the activation of cell survival pathways including PI3K/Akt, Jak/STAT, Erk and NF-kB (152). BDNF/tropomyosin receptor kinase B (TrkB) over expression in PCa is associated with increased EMT, cell migration, invasion and anoikis resistance (153). Downregulation of TrkB resulted into EMT reversal and anoikis induction (154). Antagonists of peroxisome proliferator-activated receptor-γ (belonging to the nuclear hormone receptor family), induce anoikis, disrupting the interaction of cancer cells with the ECM in squamous cell carcinoma and HCC (15, 155–157). The F-actin serving protein Cofilin that drives cell migration *via* cytoskeletal reorganization is involved in TGF-β mediated responses for progressing PCa metastasis. Functional loss of cofilin also decreases cancer cell adhesion and abolishes PCa cell invasion thus, evidence supporting (a) its role in anoikis and (b) targeting value in metastatic tumors (158).

There is a critically unmet need for a clinical tool to identify patients who can benefit from anoikis-based enhancement of therapeutic vulnerability and support clinical decision-making. Extracellular vesicles (EVs) (50-150 nm in diameter) serve as biological carriers for cargo including miRNA, proteins, lipids and anoikis effectors. Actinin-4, an exosomal protein is over-expressed in patients with CRPC or untreated metastatic prostate cancer. When the gene encoding this protein was knocked down, PCa cell growth and invasion were suppressed (159, 160). EVs isolated from the urine of prostate cancer patients have shown the presence of prostate cancer biomarkers, PCA-3 and TMPRSS2:ERG thus showing specific diagnostic and clinical value for the transcriptome within tumor EV (161). EVs carrying miRNA cargo in PCa contribute to anoikis induction *via* ECM degradation (162). Once prostate cancer specific, anoikis priming signatures are created, EVs with anoikis cargo that are present in body fluids such as blood, urine and saliva can be defined as predictive markers for PCa (163).

## Author contributions

Conceptualization: PN, NK. Investigation: PN. Original draft preparation: PN. Writing and editing: PN, NK. Supervision: NK. All authors contributed to the article and approved the submitted version.
